# A virtual research showcase and judging platform created from a patchwork of workplace applications

**DOI:** 10.5195/jmla.2022.1345

**Published:** 2022-10-01

**Authors:** Lori Gawdyda, Kimbroe Carter, Roy Morcos

**Affiliations:** 1 Lori_Gawdyda1@mercy.com, Manager, Health Sciences Libraries, St. Elizabeth Youngstown Hospital, Youngstown, OH.; 2 kjcarter@mercy.com, Librarian, Jeghers Medical Index, St. Elizabeth Youngstown Hospital, Youngstown, OH; Computer Technology, Regional College, Kent State University at Trumbull, Warren, OH; Northeast Ohio Medical Universities, College of Medicine, Rootstown, OH.; 3 Roy_Morcos@mercy.com, Associate Director, Saint Elizabeth Boardman Family Medicine Residency Program, Boardman, OH; Professor, Department of Family and Community Medicine, Northeast Ohio Medical University, Rootstown, OH.

**Keywords:** Online systems, education, medical, continuing, COVID-19, user-computer interface

## Abstract

**Background:**

Despite the challenges the COVID-19 pandemic placed on libraries' existing workflows and operations, many librarians developed and debuted new services that addressed novel needs that emerged during the pandemic. This report describes how two electronic resource librarians at regional hospitals within a healthcare corporation used exhibition platforms to showcase resident research in an online format as a complement to in-person resident research programming.

**Case Presentation:**

Over the course of the pandemic, two exhibition platform variants were implemented, one year apart. This case report describes how each platform was developed. The first online event was conducted using a virtual exhibit platform to minimize in-person contact. The second online event, held the following year, blended a traditional live event with virtual elements using the online exhibit platform. To ensure completion of tasks, project management techniques were adopted throughout the event planning process.

**Conclusions:**

The pandemic created opportunities for hospitals to explore transforming meetings from primarily live and onsite into hybrid and fully virtual events. While many corporate hospitals have transitioned back to primarily in-person programming, newly adopted online practices such as online judging platforms and automation of continuing medical education tasks will likely remain. As in-person restrictions within healthcare settings are lifted or eased at uneven rates, organizations may continue to explore the value of in-person meetings versus the video conference experience of the same meeting.

## BACKGROUND

Over the past several years, the coronavirus (COVID-19) pandemic has forced library lockdowns, work stoppages, and furloughs, impacting the daily workflows of medical librarians. During the onset of the pandemic, these challenges were felt by electronic resources librarians, who are responsible for the management of digital collections of medical books and journals. This report describes how two electronic resource librarians at regional hospitals within a healthcare corporation used this expertise in web technologies and networking infrastructure to assist in the development of an online showcasing of resident research [1], a yearly event otherwise threatened with cancellation or curtailment due to the COVID-19 pandemic. This was accomplished by utilizing software applications in the library workplace and Edublogs, a popular blog for teachers and virtual classrooms.

Prior to the onset of the COVID-19 pandemic, the regional hospitals hosted a yearly resident research day as an on-premises live event for their medical trainees. This event was a time-honored tradition that allowed residents to share the outcomes of their research as either exhibit posters or oral presentations and compete for monetary prizes and recognition. Through feedback from peers and staff, as well as interactions with judges, the residents gained invaluable experience in presenting and discussing their research results. However, the COVID-19 pandemic led to public health restrictions over the past two years, and the resident research day was threatened with cancellation.

The residency programs faculty were determined that resident research day should continue despite the established constraints on in-person gathering. However, the medical faculty lacked the expertise in online infrastructure needed to configure an online event that simulated the essence of a live occurrence. We realized that techniques used in managing digital libraries, including how to navigate tightly controlled organizational network security systems, might prove useful in creating a virtual simulation of displaying posters, delivering oral presentations, and interacting with judges [2].

## CASE PRESENTATION

Although commercial turnkey applications exist for hosting and showcasing exhibitions, their high cost exceeded our budgetary limits [3]. Hospital administrators viewed COVID-19 disruptions as transient and hoped that training activities would be normalized soon. With workforce shortages and scarcity of time necessary to evaluate a commercial application, acquiring a software package was deemed untenable. Rather, an in-house solution needed to be developed utilizing software available in the workplace or on the Internet that was priced within the departmental budget and compatible with the corporate network security.

To develop this solution, faculty educators and library staff collaborated to organize resident research exhibitions hosted in an online showcase and judging platform. An ad-hoc working group of faculty educators and electronic resource librarians was formed. The librarians, experienced in technology deployment and information technology (IT) management practices, also contributed a knowledge of navigating the corporate network security system. Working group members from medical education departments brought to the planning and design sessions knowledge of important features of a live event and views of what was needed for a successful virtual research exhibition. Requirements of the new system were to make scientific medical abstracts, posters, and oral presentations available over a corporate intranet and the Internet. Furthermore, the system needed to facilitate judging of posters and oral presentations and automate the process of producing and distributing continuing medical education (CME) certificates.

The concept of these components evolved during the committee's discussions about the event requirements, and over time an incremental build style was adopted. This development approach was patterned after agile methodologies, consisting of cycles of obtaining committee members' views, building requirements into applications, testing builds, and repeating the process until a satisfactory result was achieved [4,5]. A schematic of the basic design for the site is shown in the Figure 4.

The oral presentations formed the centerpiece of the resident research day event. During the pandemic, oral presentations on research day were hosted with Zoom. Following the last presentation, the event ended with completion of judging evaluations, tallying of the scores, and announcement of the winners. Preparation for this day started months earlier. Resident researchers submitted abstracts of their work that were evaluated by a committee with evaluations managed manually. The research was either accepted for a poster exhibit or as an oral presentation, in which case supporting PowerPoint slides were prepared. Then, these were submitted for editorial review and subsequently uploaded into the online showcase. Volunteer judges assigned to review either the abstracts or oral presentations were included in the judging platform. Over the two weeks prior to the resident research day, poster exhibits were made available for review. Judges' questions and researchers' responses could be exchanged through email or iMessage.

The assessments of oral presentations were completed on research day upon completion of the last event. Before the oral presentation, the research could be reviewed as a PDF abstract or as a PowerPoint slide deck. On research day, the content and clarity of the researcher's oral presentation could be more accurately evaluated by the judges, having previously reviewed the research in the online system.

Over the course of the pandemic, two exhibition platform variants were implemented, one year apart. Each platform required about six weeks of work. The first online event was a virtual exhibit platform, minimizing in-person contact. The goal of the second online event held the following year was to create a platform that allowed a traditional live event blended with virtual elements.

**Figure 1:**
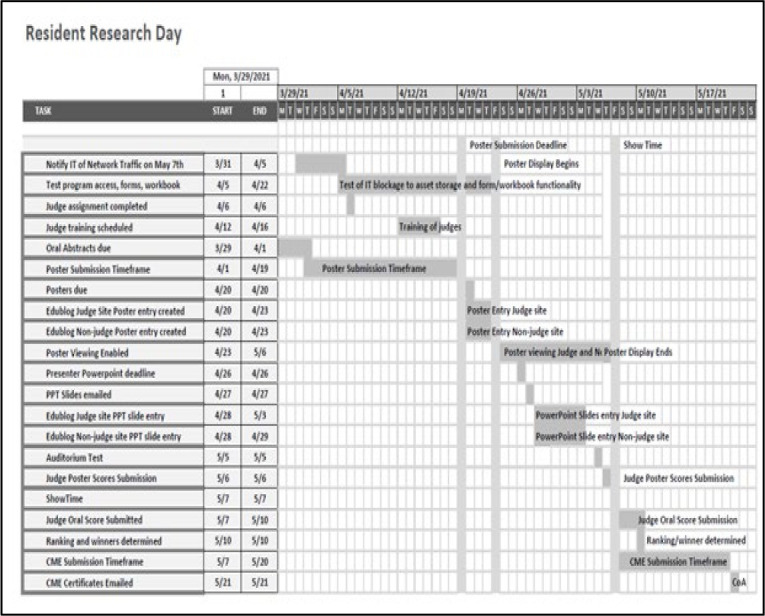
Gantt Chart

The pattern of committee work was similar for each showcase deployment. Several project management techniques were adopted to ensure the project planning met the timelines established by the group [6]. The approach helped identify dependencies and collaboration among tasks. Weekly reports listed important target objectives while milestones and progress were tracked with Gantt charts by a librarian [7, 8]. The Gantt chart used in the first deployment is shown in Figure 1 and was constructed from a Microsoft Excel spreadsheet template freely downloadable from the Internet [9]. Over the course of each project life cycle, we used the applications listed in Table 1.

**Table 1:** Applications Used in the Online Conference Development

Software Used in System Development	Function
Edublogs	Content management
LibGuides	File storage
MS Excel	Creation of the event directory
MS Forms	Judging and CME forms
MS Forms for Excel	Backend database, score tabulation
MS Word	Certificates of attendance, CME macro
MS SharePoint	File management
MS PowerPoint	Poster and presentation files
MS Outlook	E-mailing certificates of attendance
MS Teams	Access of MS Forms for Excel
Zoom	Oral presentations on research day

* MS, Microsoft; CME, continuing medical education.

While these applications are likely familiar to most medical librarians, Edublogs deserves additional attention. Edublogs is a provider of teaching website templates, which are highly configurable, designed to share information among students, and for managing an online classroom. Edublogs sites are centered around the individual teacher and students, avoiding the complexities of a large academic content management system (e.g., Canvas, Blackboard, etc.) [10]. The librarians suggested that Edublogs' capabilities for communicating information and sharing feedback could be used to create an online version of the resident research day experience. As a cloud application, Edublogs' footprint on the corporate network was small and its functionality with existing software applications was mostly unobstructed by corporate network security.

Conceptually, configurations of Edublogs and LibGuides are similar, the latter being a widely licensed web application for showcasing information about a library and its resources [11]. The Edublogs platform was readily transformable with the use of themes. Various layouts were used to display abstracts, poster submissions, and PowerPoint presentations. For the first showcase deployment, the Gridster-Lite theme was chosen for its grid-like design, a left sidebar for building navigation menus, and filtering capabilities. For the second showcase deployment the following year, the Ignite theme was chosen. While having some structural similarities to the Gridster-Lite theme, there were noticeable differences in the background colors and font.

**Figure 2:**
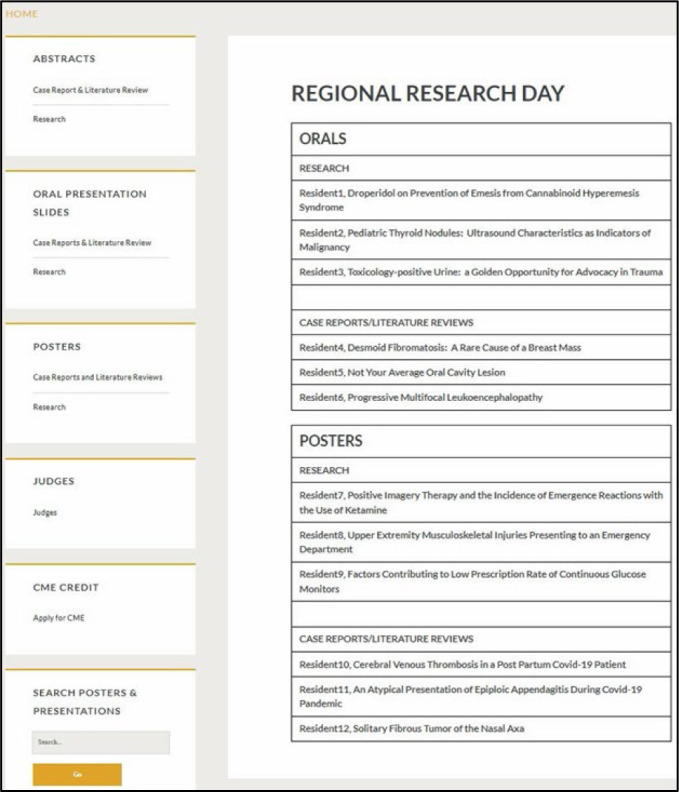
Home page of the public site

Figure 2 shows the basic layout used in the second deployment. In this figure, the space on the right was used to display a directory of exhibits. Additionally, the space was used for posters, embedded videos, and questionnaires. The sidebar on the left side of the page contained a navigation system built from hyperlinks. Selection boxes and text boxes were added to the sidebar using widgets, software components capable of enhancing functionality. These premade software elements required no coding by the librarians.

These boxes were modified to serve as filters by assigning categories to exhibit entries. For example, a judge's name was made into a category and assigned to the exhibitors' posts. When a judge's name in the sidebar dropdown for the find box was selected, only posters assigned to that judge were displayed. Exhibits were categorized into types of work, such as clinical studies, case reports, and literature reviews. This made filtering possible based on the type of work. Further refinements were made by modifying the site's cascading style sheets. These changes included the removal of the footer and resizing of the sidebar.

Exhibit posters were made in Microsoft PowerPoint [12]. These posters were standardized to a size of about 84 cm high and 120 cm wide and followed the design specifications of an academic research poster [13]. PowerPoint was also used to make slides for the oral research presentations. Once a resident's submission was approved by the training faculty, the file was converted to a PDF and sent to the library staff for uploading into LibGuides storage.

**Figure 3:**
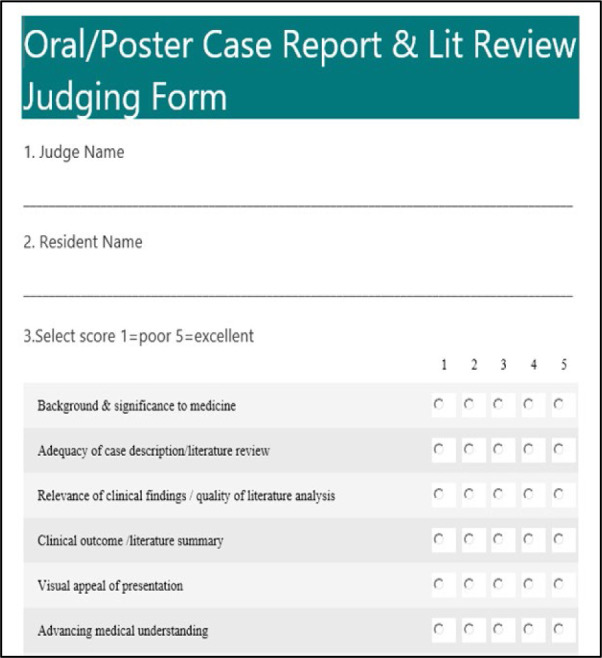


Each resident entry had an embedded judging form. Judging assessments were collected using Microsoft Forms, and results were recorded in an Excel workbook. A Microsoft Office 365 Teams application called Forms for Excel facilitated data entry through customizable forms [14]. These forms containing judges' questions and responses were stored within rows of one main Excel worksheet. In the first use of this showcase, judging forms included six textual questions and numeric Likert scales, with responses ranging from one for poor to five for excellent. An example of an oral/poster case report form is shown in Figure 3. Once submitted, the judges' evaluations were inserted into a spreadsheet. In the second showcase setup, only one question was asked within the form. By reducing the number of questions to one, the showcase planners hoped that a first impression of a poster, while perhaps relying on intuition, might result in a high level of agreement between the judges [15].

**Figure 4:**
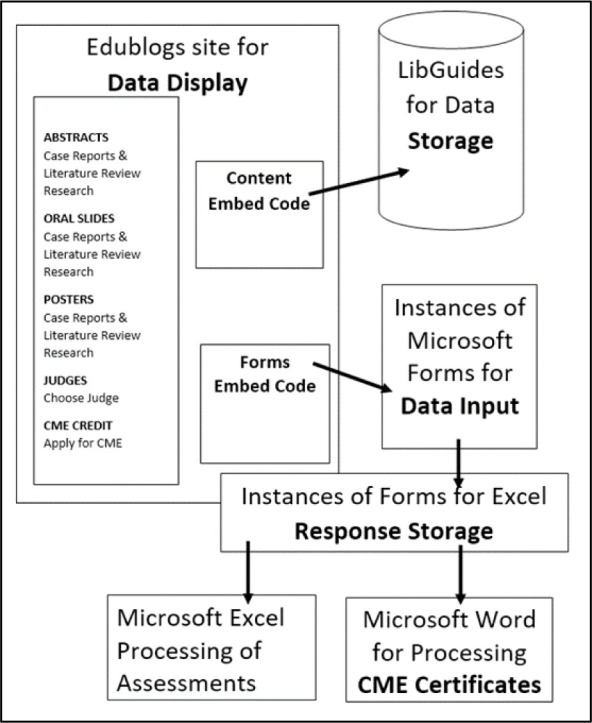
Design schematic of the showcases

In the first deployment of the showcase, two Edublogs sites were configured, one for access by the casual site visitor and the other site for judges. The judging site was password protected, thus excluding non-judges from corrupting the assessment records. In the other site for visitors, judging software components were removed and there was no link to the Excel worksheet. In the second showcase deployment, only one Edublogs site was used. Control logic, based on a password, conditionally determined enablement and display of the judging elements.

Rows in the linked worksheet held the judging responses as text or numeric Likert scores. At least four different judges evaluated each presenter. This use of Forms for Excel permitted real-time monitoring of judging activity and identification of incomplete assessments, as responses in the judging form populate the connected Excel sheet in real time. Upon completion of judging, Excel pivot tables aggregated the Likert scores and rankings, thus permitting prompt identification of the winners.

The recording of CME data was made possible using a customized form based on Forms for Excel. Responses to CME accreditation questions, the applicant's name, and email address were recorded in a way that resembled the gathering of judging assessments. An easily recognized URL was used to navigate to a CME questionnaire, which remained active for two weeks after conclusion of the event.

Upon closure of the CME site, certificates of attendance were batch processed. For the first showcase, a Word macro was developed to automate most of the clerical work of CME processing, including producing and emailing individualized certificates of attendance. The approach was restrictive in that only one desktop computer hosted the Word macro. For the following showcase, another technique was adopted using an Acrobat PDFMaker Office COM add-in to Word to process the certificates of attendance.

The basics of the two approaches were similar. A certificate of attendance template was created as a Word document [16]. Using Word mail merge and Acrobat PDFMaker add-in, the Word certificates were converted to PDFs, and individualized certificates were attached to an email and sent via the default email provider. The Acrobat add-in was not restricted to one computer. For many corporate workstations, this Acrobat add-in was part of the Word setup.

## DISCUSSION

This case report describes how a patchwork of applications can be configured into two different online showcases for displaying and facilitating judging of research exhibits. This Edublogs approach, in addition to having considerable flexibility and customizability, can be completed within four to five weeks at much lower costs than commercial, purpose-built platforms.

In its first use, the showcase and judging platform ran in full virtual mode, requiring no conference rooms, no display of printed exhibits, and no in-person attendance. In the second use, one year later, the event had a live onsite element with printed posters displayed in conference rooms, allowing in-person attendees to view and discuss them. Concurrently, an online exhibit ran, permitting remote viewing and judging. Thus, there was a blending of remote online and live onsite hosting of the event.

With an active online judging platform available, paper judging forms and clerical data entry became unnecessary. The judges directly entered their assessments through the online platform, thus avoiding clerical involvement. Importantly, judging delays could be monitored by the exhibit organizers. Since visitors and judges were not required to attend onsite, the option existed to join remotely through the online system. Whether attending remotely or live, judges were expected to submit their assessments directly through the online platform.

The development of the first online showcase presented significant challenges, as there was no available blueprint to follow. The agile design process was labor-intensive; each cycle consisted of listening to the design committee, making changes in the applications, testing upgrades on the networks, intentionally trying to crash the system, creating safeguards, and circling back to the committee. Two librarians were involved in considering various ideas and display approaches, software configurations, and testing. On the other hand, the second deployment of the system was less challenging and less labor-intensive. Only one librarian was needed to complete the second system configuration for the design committee.

The expense for the first showcase operating in virtual mode was the annual subscription cost of $75 for two Edublogs sites plus the labor contributed by two librarians over five weeks. For the second deployment, which ran in a blended mode, there was an annual subscription cost of about $35 for one Edublogs site, labor contributed by one librarian, $3,500 for the printing of posters (approximately $70 per poster for about 50 residents), and one day of labor for the set-up and tear-down of posters.

Literature searches failed to identify similar online showcasing and judging platforms as presented in this report. The use of Edublogs, Teams, and Office applications in their more traditional roles have been well described [17, 18, 19]. However, an application to accomplish an online showcase and judging platforms for research has not been previously described.

Although Edublogs was not part of the corporate collection of workplace applications, the site was not blocked as an IT vulnerability. LibGuides had prior IT approval and became the go to storage area for content of the online showcase, meeting the networking requirements of the showcase. Meanwhile, popular cloud-based storage such as Dropbox or a public OneDrive site were blocked by the corporate firewall. While the hospital's institutional SharePoint and OneDrive servers were unblocked for employees, access was not possible for non-employees. One of the showcase requirements was to allow access on the corporate network as well as the Internet, thus permitting participation of non-corporate visitors. About half of the judges were not corporate employees and had to join remotely, which emphasized the importance of designing an IT approved solution. LibGuides also were accessible from either side of the firewall but had several storage drawbacks; LibGuides limited the size of any uploaded file to 20 MB and only permitted the direct viewing of PDF and image files.

The pandemic has been a catalyst in transforming meetings from primarily live and onsite to virtual events, while also impacting the planning and organization aspects of event programming as well. Event design and planning sessions for these resident research days were conducted entirely with Zoom video conferencing. Through this process, the showcase organizers identified several additional benefits to the virtual event, including minimizing travel, facilitating the recording of judging assessments, monitoring judges' timeliness, decreasing the time needed to determine winners, and removing barriers to distributing CME credits. However, the organizers found that virtualization of the event was unnatural compared to face-to-face encounters at the live event. Virtualization, with its screen fatigue and annoying button pushing, seemed too cognitively distracting to be enjoyable and friendly. Perhaps more to the root of the unnaturalness were the missed interactions with colleagues, notably the impromptu conversations in the hallway, reassuring smile of a judge, cordial handshake, or immediacy of asking questions of the researchers and their direct responses [20].

Will resident research days be virtual going forward? The answer at our institution is clearly no. However, some online features of the judging platform and automation of CME tasks will likely remain. As in-person restrictions within healthcare settings are lifted or eased at uneven rates, organizations such as ours will continue to explore the value of in-person meetings versus the video conference experience of the same meeting, looking for ways to retain the efficiency of online meetings while regaining the camaraderie of being in the room together.

## References

[1] Gardois P, Colombi N, Grillo G, Villanacci MC. Implementation of Web 2.0 services in academic, medical and research libraries: A scoping review. Health Information and Libraries Journal. 2012;29(2):90–109. 22630358 10.1111/j.1471-1842.2012.00984.x

[2] Ma J, Stahl L, Knotts E. Emerging roles of health information professionals for library and information science curriculum development: A scoping review. Journal of the Medical Library Association. 2018;106(4):432–44. 30271284 10.5195/jmla.2018.354 PMC6148628

[3] Cost Analysis: Virtual Career Fair Vs. Physical Career Fair-vFairs.com [Internet]. [cited 2022 Jul 18]. Available from: https://www.vfairs.com/cost-analysis-of-virtual-career-fair-physical-career-fair/.

[4] Ciric D, Lalic B, Gracanin D, Tasic N, Delic M, Medic N. Agile vs. Traditional approach in project management: Strategies, challenges and reasons to introduce agile. Procedia Manufacturing. 2019;39(2019):1407–14.

[5] Chang M. An Agile approach to library IT innovations. Library Hi Tech. 2010;28(4):672–89.

[6] Burress T, Rowell CJ. Project management for digital projects with collaborators beyond the library. College and Undergraduate Libraries. 2017 Oct 2;24(2–4):300–21.

[7] Wilson JM. Gantt charts: A centenary appreciation. In: European Journal of Operational Research. 2003. p. 430–7.

[8] What Is A Gantt Chart? The Ultimate Guide – Forbes Advisor [Internet]. [cited 2022 Jul 18]. Available from: https://www.forbes.com/advisor/business/software/what-is-a-gantt-chart/.

[9] Vertex42.com. Simple Gantt Chart [Internet]. Available from: https://www.vertex42.com/ExcelTemplates/simple-gantt-chart.html.

[10] Edublogs is Enhancing the School Experience Through the Power of WordPress and Educational Technology [Internet]. [cited 2022 Jul 18]. Available from: https://www.websiteplanet.com/blog/edublogs-enhancing-school-experience-power-wordpress-educational-technology/.

[11] Ream T, Parker-Kelly D. Expanding Library Services and Instruction Through LibGuides. Med Ref Serv Q. 2016;35(3):342–9. 27391185 10.1080/02763869.2016.1189790

[12] PosterPresentations.com. Overview of the research poster templates [Internet]. Available from: https://www.posterpresentations.com/research-poster-template-overview.html.

[13] Gundogan B, Koshy K, Kurar L, Whitehurst K. How to make an academic poster. Annals of Medicine and Surgery. 2016; 11:69–71. 29225822 10.1016/j.amsu.2016.09.001 PMC5714380

[14] Forms for Excel, new experience for Excel survey in Office 365 - Microsoft Tech Community [Internet]. [cited 2022 Jul 18]. Available from: https://techcommunity.microsoft.com/t5/microsoft-forms-blog/forms-for-excel-new-experience-for-excel-survey-in-office-365/ba-p/109195.

[15] Smith P, Fuller G, Dunstan F. Scoring posters at scientific meetings: first impressions count. R Soc Med. 2004 Jul;97(7):340-1. 10.1258/jrsm.97.7.340 PMC1079533 15229266

[16] Creating a Certificate in Microsoft Word - YouTube [Internet]. [cited 2022 Jul 18]. Available from: https://www.youtube.com/watch?v=ZrEn9eCWYeI.

[17] Step 1: Set Up Your Class Blog – Courses & PD [Internet]. [cited 2022 Jul 18]. Available from: https://teacherchallenge.edublogs.org/step-1-set-up-your-class-blog/.

[18] How to enhance traditional teaching methods with Microsoft Teams [Internet]. [cited 2022 Jul 18]. Available from: https://pulse.microsoft.com/en/work-productivity-en/education-en/fa2-how-to-enhance-traditional-teaching-methods-with-microsoft-teams/.

[19] Almodaires AA, Almutairi FM, Almsaud TEA. Pre-Service Teachers' Perceptions of the Effectiveness of Microsoft Teams for Remote Learning. International Education Studies. 2021;14(9).

[20] Karl KA, Peluchette J V., Aghakhani N. Virtual Work Meetings During the COVID-19 Pandemic: The Good, Bad, and Ugly. Small Group Research. 2022 Jun 1;53(3):343–65. 10.1177/10464964211015286 PMC8165498 38603094

